# The Promise of Preventive Cancer Vaccines

**DOI:** 10.3390/vaccines3020467

**Published:** 2015-06-17

**Authors:** Pier-Luigi Lollini, Federica Cavallo, Patrizia Nanni, Elena Quaglino

**Affiliations:** 1 Department of Experimental Diagnostic and Specialty Medicine (DIMES), University of Bologna, Viale Filopanti 22, Bologna 40126, Italy; E-Mail: patrizia.nannni@unibo.it; 2 Department of Molecular Biotechnology and Health Sciences, Molecular Biotechnology Center, University of Torino, Via Nizza 52, Torino 10126, Italy; E-Mails: federica.cavallo@unito.it (F.C.); elena.quaglino@unito.it (E.Q.)

**Keywords:** cancer vaccines, cancer immunoprevention, cancer immunotherapy, Her2, immune checkpoint inhibitors

## Abstract

Years of unsuccessful attempts at fighting established tumors with vaccines have taught us all that they are only able to truly impact patient survival when used in a preventive setting, as would normally be the case for traditional vaccines against infectious diseases. While true primary cancer prevention is still but a long-term goal, secondary and tertiary prevention are already in the clinic and providing encouraging results. A combination of immunopreventive cancer strategies and recently approved checkpoint inhibitors is a further promise of forthcoming successful cancer disease control, but prevention will require a considerable reduction of currently reported toxicities. These considerations summed with the increased understanding of tumor antigens allow space for an optimistic view of the future.

## 1. Cancer Prevention: A Primer

Prevention aims at reducing cancer morbidity and mortality [1]; attempts are made to cover cancer at every step throughout its progression from normal cells to metastatic spread. Prevention is conventionally subdivided into primary, secondary and tertiary (Table 1 and Table 2).

**Table 1:** Standard definitions of prevention.

Type of prevention	Institute of Medicine of the National Academies, USA [2]	IARC, World Health Organization [3]
Primary	Primary prevention refers to health promotion, which fosters wellness in general and thus reduces the likelihood of disease, disability, and premature death in a nonspecific manner, as well as specific protection against the inception of disease.	Primary prevention is prevention of disease by reducing exposure of individuals to risk factors or by increasing their resistance to them.
Secondary	Secondary prevention refers to the detection and management of presymptomatic disease, and the prevention of its progression to symptomatic disease.	Secondary prevention (applied during the preclinical phase) is the early detection and treatment of disease. Screening activities are an important component of secondary prevention.
Tertiary	Tertiary prevention refers to the treatment of symptomatic disease in an effort to prevent its progression to disability or premature death. The overlap with treatment is self-evident, and perhaps suggests that preventive medicine has grandiose territorial ambitions. Be that as it may, there is a legitimate focus on prevention even after disease develops, such as the prevention of early cancer from metastasizing […]	Tertiary prevention (appropriate in the clinical phase) is the use of treatment and rehabilitation programmes to improve the outcome of illness among affected individuals.

**Table 2:** Types of cancer prevention.

Cancer prevention	Aim	Target	Non-immunological examples	Immunological examples
Primary	Removal or avoidance of cancer risk factors	Healthy individuals	Healthy diet; Ban on carcinogens in the workplace; Quitting smoking; Tamoxifen in healthy women; Prophylactic mastectomy in hereditary breast cancer	Anti- HBV and HPV vaccines
Secondary	Early diagnosis and therapy	Pre-symptomatic cancer bearers	Pap test; Mammography; Colonoscopy	Anti- Her2 and MUC1 vaccines against preneoplastic or early neoplastic lesions
Tertiary	Prevention of relapse and metastasis	Survivors with occult neoplastic lesions	Prophylactic radiotherapy; Adjuvant chemotherapy	Adjuvant monoclonal antibodies; Adjuvant therapeutic vaccines; Intravesical instillations of Bacillus Calmette-Guerin

### 1.1. Primary Cancer Prevention

The aim of primary prevention is the removal of risk factors from the lives of healthy individuals to, therefore, avoid cancer development altogether. This concept derives from studies of exogenous carcinogens, including occupational carcinogens and tobacco smoke [4]. Then came the idea that exposure to endogenous carcinogens could also be prevented using specific drugs (chemoprevention), such as selective estrogen receptor modulators and aromatase inhibitors, which reduce the exposure of the mammary epithelium to estrogens [5], and non-steroidal anti-inflammatory drugs, which dampen carcinogenic inflammation in the colonic mucosa [6,7].

Vaccines against carcinogenic viruses have provided a novel twist to the tale of primary cancer prevention (Table 2). The first successful example of this was the vaccine against the hepatitis B virus (HBV), which can cause chronic hepatitis, cirrhosis, and liver cancer. Pioneering studies on Taiwanese children gave a 70% reduction in hepatocellular carcinoma after the vaccination program [8]. Soon to follow were vaccines against the human papilloma virus (HPV), which is essentially a family of carcinogenic, sexually transmitted viruses that cause a spectrum of neoplastic diseases, ranging from benign lesions to metastatic carcinomas. Pre-approval trials showed very high vaccine efficacy, with a level of cancer prevention of up to 100% [9]. The widespread adoption of vaccination programs could lead, for the first time in human history, to the disappearance of a lethal carcinoma, just as vaccination led to the eradication of smallpox.

### 1.2. Secondary Cancer Prevention

Secondary cancer prevention is formed around the concept of cancer progression. Symptomatic, malignant tumors not only result from the dimensional growth of smaller lesions, but also from the progressive accumulation of multiple genetic alterations that drive a normal cell to change into a metastatic tumor. Hence, early diagnosis can uncover neoplastic lesions that are smaller and, more importantly, less advanced and more easily cured than those that are diagnosed after the onset of symptoms. Secondary prevention is implemented at the population level by means of mass screenings, such as Pap tests, mammography scans, and colonoscopy procedures [4]. Early diagnosis in itself is obviously useless without effective early therapy. Thus, to be more precise, secondary cancer prevention consists of early diagnosis followed by early therapy. In most instances, it is surgery that is used to definitively treat early neoplastic lesions discovered in early diagnosis. Where this is the case, immunoprevention is not expected to play a role. However, there is a wide range of conditions that fall on the boundary line between high-risk preneoplasia and early neoplasia, for which surgery might not be the treatment of choice and which are currently often left untreated, or are only treated with low efficiency chemopreventive agents [10]. There is currently a lack of highly effective approaches to the prevention of progression in oral leukoplakia, asbestosis, and monoclonal gammopathies, to name only a few. These and many others are potential candidates for the development of vaccines and other types of immunological secondary prevention.

### 1.3. Tertiary Cancer Prevention

Tertiary cancer prevention is actual therapy that aims to avoid tumor recurrence and metastatic dissemination. Two typical examples are prophylactic radiation treatments for breast cancer patients, to reduce the risk of local recurrence after lumpectomy [11], and adjuvant drug therapy for patients at risk of distant micrometastases after the removal of a primary tumor with unfavorable prognostic parameters [12]. As commented by Katz and Ali (see Table 1) [2], “The overlap (of tertiary prevention) with treatment is self-evident, and perhaps suggests that preventive medicine has grandiose territorial ambitions. Be that as it may, there is a legitimate focus on prevention even after disease develops, such as the prevention of early cancer from metastasizing…”. A conceptual difference between adjuvant therapy and the therapy of metastatic patients is that the former is administered on a probabilistic basis (*i.e.*, variable proportions of subjects with and without the disease receive the same treatments), whereas the latter is administered deterministically (*i.e.*, only affected patients are treated). The probabilistic element, which is a fundamental property of prevention, is the reason why preventive medicine labels adjuvant therapies as tertiary prevention.

Going beyond the theoretical issue, we found that a vaccine designed for cancer immunoprevention was ineffectual against established tumors, but prevented the growth of micrometastases [13], and we argued that the conceptual framework of preventive medicine could be appropriate to define the therapeutic limits of cancer vaccines [14].

A major problem in the development of preventive cancer vaccines is the direct translation from preclinical studies to large primary prevention trials in humans. We argued that a more feasible way would go from successful primary cancer prevention in mice to tertiary prevention (*i.e.*, adjuvant therapy) in humans, and only then to primary or secondary prevention in humans [14]. For this reason, in the present review, we discuss both immunopreventive and (selected) immunotherapeutic approaches.

## 2. Toward Cancer Immunoprevention: Lessons Learned from Preclinical Testing

The use of vaccines in the prevention of infection-associated tumors is a natural and direct consequence of the general principles of vaccination and has grown to become a cancer prevention reality [15,16,17]. The reasons why vaccination can be applied to non-infectious tumors, which make up the majority of human cancers, are perhaps less intuitive. The characterization of several tumor antigens in non-infection-related cancers [7], evidence for the fact that immune responses against these antigens are detectable in a substantial proportion of patients, and an improved understanding of the relationship between tumors and the immune system [18,19] have all provided the rationale behind and have led to the development of many sophisticated strategies for anti-tumor vaccination [20].

**Figure 1:**
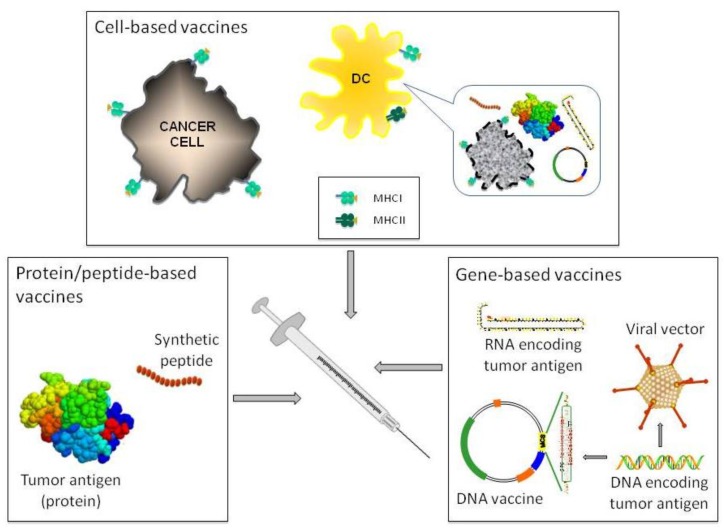
Schematic representation of the different anti-cancer vaccination strategies. The three broad types of cancer vaccines used are shown. Cell-based vaccines include cancer cells or, most often, DC pulsed or transfected with various sources of tumor antigens as depicted in the inset.

There are three broad types of cancer vaccines: cell-, protein/peptide-, and gene-based vaccines (Figure 1), and none of them are devoid of pitfalls. Cell-based vaccines can be prepared with autologous or allogeneic tumor cells [21], or most often with autologous dendritic cells (DC) pulsed or transfected with tumor antigens in various forms (*i.e.*, tumor lysates, purified proteins or peptides, DNA or RNA) [22]. However, immunogenic, cell-based vaccines have features that hamper their cost-effective, large-scale production as exemplified by sipuleucel-T odyssey (which is further examined in Section 3.3). Being molecularly defined synthetic vaccines, protein/peptide- and gene-based vaccines are more suitable for large-scale pharmaceutical manufacturing processes. However, the former display a limited immunogenicity, thus requiring the use of adjuvants. In the case of peptides, a further limitation is represented by the fact that their application is limited to patients with specific human leukocyte antigen (HLA) molecules [23]. The major drawback of gene-based vaccines is their limited uptake and consequent limited antigen transcription by transfected cells [24]. Their administration through electroporation or viral-mediated delivery solves the issue but opens new problems. In the case of electroporation, the availability of clinically approved devices and patients’ compliance have limited, until now, their use in clinic [25]. In the case of viral-mediated delivery, the problems are mainly related to potential dangers associated with the administration of live virus together with the presence of anti-viral neutralizing antibodies in patients [26].

Harnessing the patient’s own adaptive immunity to fight cancer cells without harming normal cells is, in principle, a powerful weapon with which to combat any type of cancer. Indeed, anti-tumor vaccination has a long history of success in immunization-protection experiments, and several types of anti-cancer vaccines have been successfully designed, manufactured, and pre-clinically tested [27]. However, effectiveness in inducing a measurable immune response and in extending patients’ overall survival has been modest in clinical trials. Only very recently have promising results from a handful of clinical trials modified the cancer vaccination landscape [20], which is now entering a new era [28].

The discrepancy between the efficacy of vaccines in preclinical experiments and clinical trials is actually deceptive, as a careful analysis of preclinical data would have predicted clinical failure in most cases. In retrospect, we now know that the experimental results that have led to the enthusiastic clinical application of many anti-cancer vaccines showed bias, since most of these experiments were performed by injecting transplantable cancer cell lines into young and healthy syngeneic mice that did not present the cancer-induced constraints on immune response and the central and peripheral mechanisms of tumor antigen tolerance [29,30,31]. Moreover, the high proliferation rate of transplantable tumor cells does not reproduce the architectural and cellular complexity of real cancers and thus minimizes the consequences of tumor genetic instability, immune editing, and tumor escape ability [32,33,34].

### 2.1. Genetically Modified Mouse Models

The advent of genetically modified mice (GEM) engineered to express oncogenes, or in which tumor suppressors have been disrupted, and spontaneously develop tumors, has revolutionized preclinical cancer research. Despite still having a few limitations, these mice are a realistic model that can predict the effectiveness of anti-cancer vaccines as the relationships between the incipient tumor, the immune system, and the surrounding tissues are preserved, while carcinogenesis progression can mimic what is observed in humans, including metastases development [32].

Results from several preclinical anti-cancer vaccination experiments in various GEM models have clearly shown that the elicited immune response and the efficacy of anti-tumor protection against autochthonous tumors becomes progressively lower as vaccination is started at later stages of carcinogenesis [13,14,27,35,36].

An example is provided by experiments in BALB-neuT mice. These mice are transgenic for the mutated form of the rat ortholog of the human epidermal growth factor receptor 2 (Her2) oncogene under the transcriptional control of the mouse mammary tumor virus promoter and develop mammary carcinomas with well-defined progression, which resembles that of human breast tumors in many aspects [37,38,39]. When BALB-neuT mice are vaccinated against Her2, using either cell- [40,41,42,43] or gene-based vaccines [27,30,35,44,45,46,47,48], the intensity and effectiveness of the induced immune response are inversely proportional to the stage of carcinogenesis progression at which vaccination is started. Mice bearing precancerous lesions were successfully protected with anti-Her2 vaccination alone. However, significant protection against microscopic neoplastic lesions was only achieved when anti-Her2 vaccination was associated with protocols that contrast tumor-induced immune suppression, such as T regulatory cell depletion [13,49], or when vaccination was directed against the tumor vasculature, as in vaccination against angiomotin, which is one of the angiostatin receptors expressed by endothelial cells in BALB-neuT tumors [50]. These data thus demonstrate that cancer prevention is very much an obtainable goal [51,52].

Down-modulation of major histocompatibility complex expression in murine [53] and human [54] tumors frequently hampers cytotoxic T cell (CTL) recognition. Mechanistic studies in Her2 transgenic mice revealed a strong contribution of helper T cell cytokines and anti-Her2 antibodies to cancer immunoprevention [35,40,42,46,47,48,55,56,57]. The different mechanisms at work in cancer prevention and therapy are reminiscent of viral immunity, in which infection is resolved by CTL, whereas prevention from re-infection is dependent on antibodies [14]. This parallelism with viral immunity [58] is further confirmed by the observation that transfer of anti-Her2 antibodies from immunized mothers to their cancer-prone BALB-neuT offspring delays mammary cancer progression [59].

### 2.2. Companion Animals

Another important lesson on the potentialities, and limits, of cancer vaccines can be found in their application in companion animals that bear naturally occurring tumors. Over the last decade, it has been recognized that naturally occurring tumors in cats and dogs mirror those in humans and are thus a powerful translational tool [60]. Effective vaccines against feline leukemia virus have been developed and are currently used in cats, but they can be associated with the development of vaccine-associated sarcomas at the site of injection that require aggressive surgery and, often, chemotherapy [61]. Therefore, testing of anti-cancer vaccines for translational purposes in cats must be done with caution. By contrast, this type of side effect is not seen in dogs that consequently have been more extensively used as a translational model for the development of anti-cancer vaccines. Results from a pioneering trial of anti-tyrosinase vaccination in dogs affected by malignant melanoma [62] support the idea that only patients with local tumor control and no evidence of metastasis before vaccination can benefit from immunotherapy. This idea has been corroborated by the following studies [63,64] that were carried out using a vaccine that was approved by the United State Department of Agriculture in 2009 and is now commercialized as Oncept (Merial). Significant lengthening of overall survival in dogs with locally controlled stage II-III oral malignant melanoma that expresses chondroitin sulphate proteoglycan (CSPG) 4 has recently been achieved using an anti-CSPG4 DNA vaccine [65], further demonstrating that immunotherapy is effective against minimal residual disease.

## 3. Human Cancer Immunoprevention

The data from “classic” mouse preclinical models, together with those from comparative oncology models, clearly show that only animals bearing precancerous lesions, incipient tumors, and those that are at risk of developing recurrences and metastases after primary tumor removal are protected, meaning that anti-cancer vaccines are effective only when used in prophylactic settings, *i.e.*, primary, secondary and tertiary prevention, as in the original concept of vaccination. If we transfer this concept to the human setting, it becomes clear that the application of immunotherapy regimens to the treatment of patients with advanced cancer is suboptimal. However, anti-cancer vaccines show significant potential if used to prevent tumor growth in healthy patients at a “high-risk” of developing a cancer—*i.e.*, people with occupational exposure to carcinogens [66], or hereditary mutations in breast cancer (BRCA) genes [67], or in p53 [68], *etc*.—and when treating recurrence and metastasis in cancer patients with minimal residual disease.

In this context, it must also be considered that cancer prevention is intended for healthy individuals, therefore, the lack of toxic side effects is as important as efficacy, whereas in cancer therapy, some degree of toxicity is acceptable. A case in point is the toxicity of cancer vaccines and adjuvants; therapeutic vaccines and adjuvants can cause local and systemic toxicities that would be forbidden in prophylactic vaccines. Vaccines currently used for primary cancer prevention, such as those against HBV and HPV, only cause low-grade toxicities. The same considerations apply to some of the vaccines and adjuvants that we will discuss in the following sections. Promising approaches tested in mice and in therapeutic clinical trials, such as immune checkpoint inhibitors, will not find application in human cancer prevention unless their toxicity is greatly reduced.

### 3.1. Primary Immunoprevention: A Futuristic Option for Non-Infection Associated Tumors?

The potential of primary prevention in non-infection-associated tumors was already at the top of the tumor immunology top ten list in 2008 [52], however, for obvious reasons, its application is still confined to preclinical experiments. Nevertheless, this idea has recently paved the way for the foundation of the Artemis Project, whose (perhaps overoptimistic) goal [69] is to stop people dying of breast cancer by 2020 (http://www.breastcancerdeadline2020.org). This project, supported by the US National Breast Cancer Coalition, is based on the identification of breast cancer-specific neoantigens that are expressed in the early phases of carcinogenesis and against which prophylactic vaccines of various types can be generated.

### 3.2. Secondary Immunoprevention: A Future Option Whose Efficacy Is Being Tested Now

On ClinicalTrials.gov there are more than 1600 cancer vaccine trials listed. Some of them are examples of secondary immunoprevention and are discussed here (Table 3). Recently, a pilot trial (ClinicalTrials.gov identifier: NCT00107211) has seen 38 patients with *in situ* Her2-expressing ductal carcinoma (DCIS) being vaccinated one month before lumpectomy with autologous DC that were pulsed with six HLA class II promiscuous-binding peptides from Her2 (DC1 vaccine) [70]. Vaccination was well-tolerated and induced a strong and long-lasting T cell response against Her2 in all but two treated patients and was independent from estrogen receptor (ER) and Her2 magnitude of expression. Nevertheless, the kind of clinical response observed at the time of lumpectomy in patients with ER positive and ER negative tumors was different, with the former displaying loss of Her2 tumor expression and the latter complete tumor regression [70]. In the future, we will discover whether the induced immune response has an impact on the risk of developing breast cancer events.

In another recently concluded trial (NCT00773097) of secondary immunoprevention, patients with a history of advanced adenomas of the colon, which are a precursor to colon cancer, were vaccinated with a mucin1 (MUC1) 100-mer peptide admixed with the toll like receptor (TLR) 3 agonist Hiltonol [71]. An anti-MUC1 IgG response and long-term memory were observed in about 50% of vaccinated patients, without significant adverse events. The lack of detectable immune response in the other 50% of patients was correlated with an expanded number of circulating myeloid-derived suppressor cells (MDSC) before vaccination [72]. The monitoring of cancer occurrence in vaccinated patients in the coming years will allow us to evaluate the prophylactic potential of this MUC1 peptide vaccine in preventing colorectal cancer. A new randomized, placebo-controlled phase II efficacy trial (NCT02134925) with the same vaccine is now recruiting patients with colon adenomas; results are expected by 2020 [73].

### 3.3. Tertiary Immunoprevention: The Present Option

Aside from these few examples of secondary prevention, the vast majority of cancer vaccines that have emerged from successful preclinical testing have been translated into clinical trials of tertiary cancer prevention (adjuvant therapy); here we discuss some of the more promising vaccine trials (Table 3).

**Table 3:** Cancer vaccine trials cited in the text.

ClinicalTrials.gov Identifier	Type of vaccine	Patients with:	Status
NCT00107211	Autologous DC pulsed with HLA class II promiscuous-binding peptides from Her2 (DC1 vaccine)	Her2+ breast DCIS	Completed
NCT00773097	MUC1 100-mer peptide with Poly-ICLC	Advanced colorectal adenoma	Completed
NCT02134925		Advanced colon polyps	Recruiting
NCT01431391	Autologous DC pulsed with the fusion protein PA2024 (sipuleucel-T)	Castration refractory metastatic Prostate cancer	Completed
NCT00639639	Autologous DC pulsed with CMV pp65-LAMP mRNA	Glioblastoma multiforme	Active, not recruiting
NCT00524277	Her2-derived HLA class I peptide (GP2) with GM-CSF	Her2+ breast cancer	Active, not recruiting
NCT00841399	Her2-derived HLA class I peptide (E75) with GM-CSF	Her2+ breast cancer	Completed
NCT00854789			Completed
NCT01479244			Active, not recruiting
NCT01510288	GM-CSF-transfected allogeneic prostate cancer cells	Castration refractory metastatic Prostate cancer	Terminated
NCT01417000	GM-CSF-transfected allogeneic pancreatic cancer cells and CRS-207	Metastatic pancreatic Adenocarcinoma	Active, not recruiting
NCT02004262			Recruiting
NCT02243371			Recruiting
NCT00077532	gp100-derived HLA class I peptide	Advanced melanoma	Completed

The odyssey of the first Food and Drug Administration (FDA)-approved therapeutic cancer vaccine, sipuleucel-T (Provenge), is quite an interesting example. This DC-based vaccine was approved for the treatment of asymptomatic or minimally symptomatic metastatic castration-refractory prostate cancer patients in 2010 [74]. To make sipuleucel-T, the patient’s mononuclear cells are sent to a production plan to be pulsed with a proprietary fusion protein (PA2024), which combines the antigen prostatic acid phosphatase with granulocyte macrophage colony stimulating factor (GM-CSF). Cells are then sent back to the originating center for re-infusion into the patient. The first problem with this vaccine is the cost, too expensive to justify its use by National Health Service [75]. Secondly, the therapeutic benefit in term of survival, about four months *versus* placebo, was modest in this trial. Furthermore, it was argued that the benefit “could be the result of a flaw in the trial design or from the chance imbalance of unmeasured prognostic variables” [76,77,78,79]. Dendreon, the company making Provenge, had to file for Chapter 11 and the vaccine has recently been acquired by Valeant Pharmaceuticals [75]. Immediately after, the results of the interim assessments of cellular and humoral responses through 24 months from the STAND randomized, phase II trial (NCT01431391) were announced [80], showing that Provenge is effective in inducing a robust immune response in men with biochemically recurrent prostate cancer. However, data on the therapeutic efficacy are not yet available, making the sipuleucel-T odyssey an ongoing story.

Indeed, it is widely recognized that several factors contribute to limiting the efficacy of sipuleucel-T and the other DC-based vaccines [81]. The reduced ability of the cells in the vaccine to reach the lymph nodes is among these limiting factors. Pre-conditioning of the vaccine injection site with the tetanus/diphtheria (Td) toxoid has been shown to be effective in enhancing vaccine DC lymph node homing and their ability to stimulate tumor-antigen specific T cell responses in mice [82]. Thanks to these results, Mitchell and colleagues have recently conducted a randomized and blinded clinical trial (NCT00639639) for the treatment of patients affected by glioblastoma multiforme [82]. In this study, 12 patients that were receiving chemotherapy after tumor removal were vaccinated monthly with autologous dendritic cells pulsed with Cytomegalovirus (CMV) phosphoprotein 65 (pp65)-lysosomal-associated membrane protein (LAMP) mRNA, which is expressed in 90% of glioblastoma tissues, but not in normal brain tissue. Patients received either autologous non-pulsed DC (as control) or Td at the vaccine injection site prior to vaccination, with or without autologous lymphocyte transfer. Despite the aggressiveness of glioblastoma multiforme, patients receiving Td before pp65 pulsed DC displayed a significant increase in both progression-free and overall survival [82] as compared to patients whose vaccine site pre-conditioning included only the injection of autologous DC. The clinical benefit observed in this small but very promising clinical trial was associated with a significantly higher accumulation of injected DC at the vaccine site draining lymph nodes and a higher, longer-lasting specific pp65 T cell response.

Data from another promising trial of tertiary immunoprevention were presented at the American Society of Clinical Oncology Breast Cancer Symposium in September 2014. In this randomized phase II trial (NCT00524277), breast cancer patients (any Her2 and ER expression) with no evidence of disease after completing standard treatments, including trastuzumab (Herceptin), either received the Her2-derived HLA class I peptide, called GP2, in combination with GM-CSF, or GM-CSF alone [83]. The vaccine significantly reduced the risk of recurrence and, as expected, patients with Her2^+^ tumors benefited most from the vaccination [83]. However, it is worth noting that no definitive conclusion can be drawn from this trial as the number of Her2^+^ patients was relatively small (48 and 50 in the vaccinated and control group, respectively) and the balancing of the two arms of the trial, in terms of tumor size, hormone and nodal status, should have been more carefully evaluated.

An even more promising vaccine against Her2, which has been tested in phase II clinical trials (NCT00841399, NCT00854789), is Neuvax. This is an HLA-A*0201-restricted immunogenic Her2 nonapeptide which is intradermally injected in combination with GM-CSF [84]. Patients with breast tumors expressing any degree of Her2 enrolled for the trial received standard of care therapy and were confirmed to be disease-free prior to enrollment. The vaccine was administered once a month for six months and was followed by booster shots once every six months thereafter. Disease-free survival (DFS) at five years was 89.7% in the vaccinated group *versus* 80.2% in the control group. Unexpectedly, vaccinated patients with Her2 low-expressing tumors were those that displayed the better DFS numbers [84,85]. This paradoxical result remains still unexplained. A randomized, multicenter phase III study (NCT01479244) for patients with early-stage, lymph-node-positive breast tumors that express low to intermediate levels of Her2 started in 2012 and aimed to confirm efficacy and safety in a larger population. Even if results are not yet available, nevertheless, this ongoing trial is currently the most advanced step in the development of a vaccine strategy to prevent breast cancer recurrence.

As it capitalizes on this wealth of information, cancer immunoprevention is now revolutionizing the way we treat cancer and offers unprecedented opportunities for improving the management of cancer patients on a rational basis.

## 4. Checkpoint Blockade as a Biological Adjuvant for Cancer Immunoprevention: Work in Progress

Established, growing tumors are strongly immune-suppressive and give the organism little chance to induce effective and long-lasting immunity against self-tolerated molecules such as tumor antigens. Finding appropriate strategies for counteracting tumor-induced immune suppression would allow for the successful application of cancer vaccines in therapeutic settings.

Several inhibitory pathways that contribute to tumor-induced immunosuppression (cytokines, suppressive cell population, amino acid-catabolizing enzymes, and ligation of inhibitory receptors on activated T cells) have been identified and their blockade in cancer patients is under investigation. The two most promising strategies used so far are the administration of low doses of chemotherapeutics at short intervals (metronomic chemotherapy) [86] and the administration of monoclonal antibodies (mAbs) directed against inhibitory molecules of the immune system [87].

Besides exerting antiangiogenic activity, metronomic chemotherapy stimulates anti-cancer immune responses by selectively eliminating T regulatory cells and MDSC [88]. Thus, it is a good candidate for use together with cancer vaccines for the therapy of various tumor types [89].

Several mAbs directed against inhibitory receptors have been generated and tested in preclinical models. These include mAbs against cytotoxic T lymphocyte-associated protein 4 (CTLA-4) [90,91]; programmed cell death 1 (PD-1) [92,93] and its ligand (PD-L1 or B7-H1) [92,94]; members of the killer cell immunoglobulin-like receptor (KIR) family [95]; tumor necrosis factor receptor superfamily member 4 (TNFRSF4 or OX40) [96,97,98]; TNFRSF9 (CD137 or 4-1BB) [99,100]; TNFRSF18 (GITR) [101]; and the transforming growth factor β1 (TGFβ1) [102]. Most of these mAbs have also been tested in patients bearing differing forms of solid cancer. However, only anti-CTLA-4 and anti-PD-1 mAbs have been FDA-approved for use in the clinic [22]. They are the fully human antagonistic anti-CTLA-4 mAb, ipilimumab (Yervoy), the fully human (nivolumab; Opdivo) and the humanized (pembrolizumab; Keytruda) programmed death receptor-1 (PD-1)-blocking mAbs.

**Figure 2:**
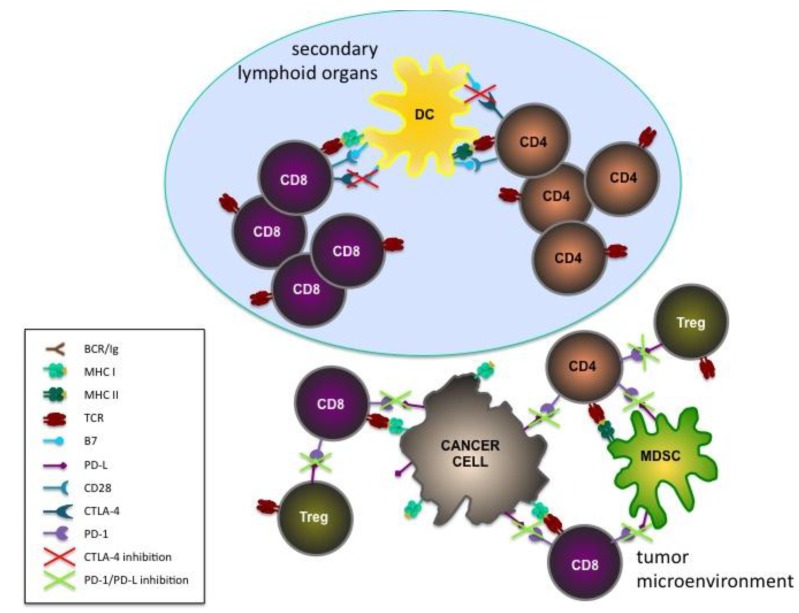
Schematic representation of the basic principles of checkpoint inhibition. The potential roles of the inhibition of CTLA-4 during the priming of T cells in secondary lymphoid organs and of PD-1 and its ligands (PD-L) during the effector phase in the tumor microenvironment are shown.

CTLA-4 and PD-1 inhibitory checkpoint pathways act in temporally and spatially distinct ways in regulating T cell response (Figure 2). CTLA-4 is mainly involved in the priming phase in secondary lymphoid organs, while PD-1 dampens the effector functions of already activated T cells in the periphery [103].

Ipilimumab was the first immunotherapeutic drug which was able to induce long-term durable responses and improve overall survival in patients with metastatic melanoma [104,105,106]. As such, it was licensed by the FDA for use in patients with unresectable advanced metastatic melanoma in 2011 [107]. In 2014, the FDA licensed pembrolizumab [108] for the treatment of metastatic melanoma patients who do not respond to ipilimumab [109] or BRAF inhibitors [110]. Pembrolizumab is now being tested in phase II/III trials in non-small-cell lung cancer (NSCLC) patients with oligometastatic disease [111] and as a monotherapy and combination therapy in 30 different cancer subtypes [112]. Nivolumab was FDA-approved for the treatment of advanced melanoma in December 2014, following the publication of results from a completed Phase III clinical trial demonstrating a significant improvement in progression-free and overall survival in patients with melanoma without the BRAF mutation [113]. On March 4th, 2015, Nivolumab also received FDA approval for the treatment of NSCLC patients as a recent phase III trial (NCT01642004) was stopped ahead of schedule because it had already met its endpoint: superior overall survival in nivolumab-treated patients compared to the standard therapy control arm [114]. The checkpoint inhibitors nivolumab and ipilimumab had demonstrated a synergic effect when given in combination to patients with advanced melanoma [115], and gave a two-year survival rate of about 80% [116]. The combination also resulted in increased side effects as compared to therapy with either agent alone; however, most side effects were still manageable and reversible, and similar to those experienced with ipilimumab alone [116]. Other ongoing clinical trials in patients with other tumor types are also providing encouraging results [117].

The effective elimination of a tumor requires coordinated immune mechanisms involving both the activation of immune effector cells and the removal of suppressor mechanisms. Therefore, there is a strong rationale for combining anti-cancer vaccines with either checkpoint inhibitors or metronomic chemotherapy [118]. However, only a few combination immunotherapy trials have been reported to date [119,120,121], some of which are listed in Table 3. Nevertheless, none of them incorporate single agent control arms, therefore conclusions on the possible additive/synergistic effects of the combination therapies are impossible to make.

A GM-CSF gene-transfected allogeneic tumor cell vaccine (GVAX), which is based on prostate cancer cells given in combination with ipilimumab, has recently been shown (NCT01510288) to be safe and tolerable in patients affected by metastatic castration-resistant prostate cancer that had not been previously treated with chemotherapy [119].

In a multicenter randomized phase II trial (NCT01417000), patients with metastatic pancreatic adenocarcinoma were treated with a pancreatic GVAX in combination with low doses of cyclophosphamide and a recombinant live-attenuated double-deleted *Listeria monocytogenes* secreting pancreatic adenocarcinoma tumor antigen, mesothelin (CRS-207) [122]. Extended overall survival as well as the induction of higher numbers of mesothelin-specific CD8 T cells were both observed in patients treated with the combination therapy as compared to those only treated with GVAX and cyclophosphamide [120]. Based on these results, a randomized, controlled, and three-arm trial will evaluate the safety, immune response, and efficacy of the combination immunotherapy of pancreatic GVAX, low-dose cyclophosphamide, and CRS-207 as compared to chemotherapy or CRS-207 alone (NCT02004262). In addition, since the FDA has designated CRS-207 and pancreatic GVAX combination therapy a breakthrough therapy, it will now also be evaluated in combination with the checkpoint inhibitor nivolumab in patients with metastatic pancreatic cancer (NCT02243371). Results are expected in January 2019.

A phase III clinical trial combining a gp-100 peptide vaccine with ipilimumab was performed in patients affected by advanced metastatic melanoma [121]. Ipilimumab, whether used with or without the vaccine, improved overall survival as compared to gp100 alone. Severe adverse events were observed, but most were reversible with appropriate treatment.

These initial results allow space for an optimistic view of the future. We have promising tools with which to fight several types of cancers, including melanoma, glioblastoma, NSCLC, breast, prostate, pancreatic, and colon cancer. However, they now need to be studied in depth for us to find the best combinations for use in cancer patients at various stages of their disease.

## 5. Target Antigens for Cancer Immunoprevention: Drivers or Passengers?

The analysis of tumor genomes is currently creating new opportunities in many sectors of oncology, including the study of tumor antigens. Molecular studies have discovered most tumor antigens that are commonly expressed by human tumors over the last century, but have barely scratched the surface when it comes to individual antigens, *i.e.*, those distinctive tumor antigens that derive from random mutations in each patient and that are potentially unique to his/her tumor.

Tumor immunologists are currently quite similar to the theoretical physicists who predicted the existence of a class of particles: we knew that individual tumor antigens existed, but now, thanks to DNA sequencing, we are able to recognize them experimentally and to devise specific therapeutic strategies. Presently, “therapeutic” is the operative word as the application of individual neoantigens to cancer immunoprevention is not yet a reality. However, sequencing could be translated into new approaches to secondary and tertiary immunoprevention in which individual tumor antigens, discovered in preneoplastic, early neoplastic, or primary lesions, can then be targeted with specific vaccines [123,124].

One of the fundamental issues here is the relationship between neoantigens and neoplastic transformation. Mutations in cancer cells are commonly divided into “drivers” and “passengers”. The former encompasses all mutations in cancer genes that cause and sustain tumorigenicity. However, the mutagenic processes that cause driver mutations invariably produce a large number of random passenger mutations, which do not contribute to the neoplastic phenotype [125].

From an immunological point of view, both driver and passenger mutations are equally interesting, as long as they give rise to recognizable tumor antigens. Great interest currently surrounds passenger tumor antigens because patients that are responsive to non-antigen specific immunotherapies, or to antigen-specific therapies against previously unknown specificities, were recently shown to express and recognize this type of neoantigen [126,127].

Returning to cancer immunoprevention, we must consider that passenger tumor antigens can be intrinsically less persistent than antigens that derive from mutations in driver cancer genes [128], because antigens that are unrelated to the growth and spread of cancer cells will easily be lost, or down-modulated, in the presence of an immune response. We coined the term “oncoantigens” to distinguish persistent tumor antigens that are directly or indirectly related to the survival, growth, and spread of tumor cells [14].

Thus, a number of different scenarios can be envisaged where the choice of antigens is matched with the type of cancer immunoprevention.

For pure primary cancer immunoprevention in healthy individuals, the governing rule is *primum non nocere,* hence, vaccines will be necessarily directed against oncofetal and “retired” antigens, *i.e.*, molecules that are no longer expressed in the individual at risk, but are likely to be expressed in tumors [129]. If such molecules are not oncoantigens, it remains to be determined whether nascent tumors can easily give rise to antigen-negative variants or not. For groups of high-risk individuals (*e.g.*, asbestos exposure, or BRCA1/2 carriers), the risk factor itself may be predictive of a specific constellation of tumor antigens, while vaccine side effects can be tolerated as the risk of carcinogenesis approaches certainty.

In secondary immunoprevention, an in-depth “omic” analysis of existing preneoplastic or early neoplastic lesions could reveal the presence of individual antigens, in addition to those that are commonly expressed [51]. The need for long-term immunity against the continuous risk of neoplastic transformation will orient the formulation of vaccines against persistent tumor antigens.

For tertiary cancer immunoprevention, the bulk of molecular information will come from the analysis of the primary tumor. Vaccines against antigens that are encoded by passenger mutations will find their best application in adjuvant immunotherapy because the immune-mediated attack on micrometastatic foci may be sufficiently rapid and destructive to prevent the generation of antigen-loss clones [130].

## 6. Conclusions

Vaccines against HBV and HPV effectively prevent hepatocellular and cervical carcinomas. Preclinical evidence shows that vaccines can also prevent tumors unrelated to infectios agents, which are the majority of human tumors. Early clinical trials are now translating immunoprevention to humans at risk of breast and colorectal cancer. The definition of optimal target antigens and the inhibition of immune checkpoints can enhance the efficacy of preventive cancer vaccines and pave the way to a broader application of cancer immunoprevention.
